# Robust MFC anti-windup scheme for LTI systems with norm-bounded uncertainty

**DOI:** 10.1038/s41598-021-81036-7

**Published:** 2021-01-21

**Authors:** Xiao-Qin Mo, Mi Zhou, Yuan Wang, Shang-Jia Guo

**Affiliations:** 1 Institute of Science and Technology, University of Sanya, Sanya City, 572000 Hainan Province China; 2 Haikou Power Supply Bureau, Hainan Power Grid Company, Haikou City, 570100 Hainan Province China

**Keywords:** Energy science and technology, Engineering

## Abstract

On the basic of the fact that all signals in the practical system are always bounded, this paper proposes a 4-degree-of-freedom (DoF) anti-windup scheme for saturated systems with parametric uncertainty. A fairly straightforward tuning rule is introduced to the robust stability analysis for the proposed anti-windup structure under the framework of IQC (Integral Quadratic Constraint). And the sufficient stability conditions are derived to check the reasonable definiteness of the related transfer function. Moreover, the control design for disturbance response and set-point tracking response are two separate part in this proposed scheme. Numerical example demonstrates the effectiveness and the considerable performance improvement of the anti-windup compensator that is designed by the proposed technique.

## Introduction

Model uncertainty and actuator saturation are two problems that control engineers often encounter. Especially in the case of model uncertainties, the problem of ensuring robustness has occupied the control field for many years. Many researchers spend a lot of time studying actuator saturation^1,2^. However, the uncertainty of the model is usually subjected to neglect in the study of actuator saturation system. This has been the case particularly with the anti-windup community, where the assumed conditions seem to make the anti-windup compensated constrained system obtain robustness from its unconstrained counterpart^3–6^ . This makes some intuitive sense, although it is reasonable to hypothesise the importance of nominal linear robustness, but not sufficient condition for the robustness of the overall anti-windup compensated nonlinear system^7^. For example, the static anti-windup compensation method in^8^, the full-order anti-windup compensation method in^9^ and the high-gain anti-windup compensation method in^10^ can all yield a better performance without considering uncertainty, but a worse robustness or even more drive the system unstable when considering uncertainty^7,8^.

Perhaps the most comprehensive explication is given in^11^, which contains a collection of papers that how to deal with the uncertain in a systematic way. However, most of these papers choice the one-step solutions where the uncertainty will be considered at the beginning of the design, but not the two-step anti-windup approach. And a particular type of uncertainty is often used, likes normally parametric or state-space uncertainty, but it is quite limited in scope in practice and not effect on capturing unmodeled dynamics^7^. In^7,12^, the authors consider the robustness of the Weston and Postlethwaite anti-windup scheme^13^ to additive norm-bounded uncertainty (which has shown itself close to the uncertainty that often used in practice). In particular, a sufficient robust stability condition of the Internal Model Control (IMC) is derived in^7^ when there is no saturation restriction in the control system. And it shows that the IMC is optimally robust to preserve the robustness of the unsaturated loop. In^14^, more general uncertainty structure is considered and the authors also investigate the stability robustness of the Weston and Postlethwaite anti-windup scheme. Based on the Integral Quadratic Constraint (IQC) theory, a sufficient robustness condition is derived and it is proven to be less conservative than existing results in the literature for additive uncertainty. Actually, IQC framework of^15^ is a perfect solution to the robustness problem of anti-windup systems with norm-bounded uncertainty and a relevant research is discussed in^16^. In^17^, a general formula of the uncertainty is presented in control systems based on the Keldysh-Green’s function formalism in the gauge-covariant Wigner space. And the more information can be found in^18–22^.The robustness analysis results of our work are also constructed under the IQC framework, the main results are provided in “Preliminaries” section.

From another perspective, actuator saturation can be regarded as a particular type of uncertainty of the unconstrained system, and hence anti-windup compensation can be treated as stability robustness problem of the corresponding unconstrained system. Among various control techniques in literature, model following control (MFC) structure is commended due to its simplicity and high robustness^23,24^ although it attracts little attention by researchers except in field of position servo systems with electrical machines^23–25^. Taking advantage of the merit of MFC structure, it is expectable to develop MFC-based anti-windup scheme so as to improve performance of anti-windup systems and to counteract the aforementioned disadvantages of modern anti-windup techniques.

This paper is structured as follows. Some preliminaries regarding IQC and structure of MFC are presented in “Preliminaries” section. In “Proposed anti-windup scheme based on MFC” section, the proposed anti-windup scheme is developed and some attractive properties of the proposed anti-windup scheme are discussed; robustness analysis of the resulting system is presented and sufficient condition is derived on the basis of IQC framework in “Robustness analysis” section, and hence the tuning rule for robust stability is obtained. In “Conclusions” section, numerical example illustrates the remarkable robustness improvement of the proposed anti-windup scheme with the comparison of three existing anti-windup techniques.

## Preliminaries

Consider linear-time-invariant (LTI) ${\mathrm{n}} \times {\mathrm{n}}$ plant

1 $$G(s) = \left\{ {\frac{{B_{\mathrm{ij}} (s)}}{{A_{\mathrm{ij}} (s)}}} \right\},\quad {\text{i}} ,\;{\text{j}} = 1, \ldots , {\text{n}}$$

or

2 $$G(s) = \frac{B(s)}{{A(s)}}$$

for SISO system, where $A_{ij} (s)$ and $B_{\mathrm{ij}} (s)$ or $A(s)$ and $B(s)$ are real coprime polynomials. In many cases^8,13^, it is presented by state space realization

3 $$G(s)\sim \left\{ {\begin{array}{*{20}l} {\dot{x}_{{\text{p}}} = A_{{\text{p}}} x_{{\text{p}}} + B_{{\text{p}}} u + B_{{{\text{pd}}}} d} \\ {y = C_{{\text{p}}} x_{{\text{p}}} + D_{{\text{p}}} u + D_{{{\text{pd}}}} d} \\ \end{array} } \right.$$

where $x_{p} \in {\mathbb{R}}^{{{\mathrm{n}}_{{\mathrm{p}}} }}$ is the plant state, $u \in {\mathbb{R}}^{{\mathrm{n}}}$ is the control input, ${d} \in {\mathbf{\mathbb{R}}}^{{{\mathrm{n}}_{\text{d}} }}$ is the exogenous disturbance input, ${y} \in {\mathbf{\mathbb{R}}}^{{\mathrm{n}}}$ is the plant output available for measurement, and $A_{{\text{p}}}$, $B_{{\text{p}}}$, $C_{{\text{p}}}$, $D_{{\text{p}}}$, $B_{{{\text{pd}}}}$ and $D_{{{\text{pd}}}}$ are real constant (or matrices with suitable dimensions)^26^. The control input ${u}_{\mathrm{i}}$ is constrained such that

4 $${u}_{\min }^{\text{i}} \le {u}_{\mathrm{i}} \le {u}_{\max }^{\text{i}} ,\quad {\text{i}} = 1, \ldots , {\mathrm{n}}.$$

and that is represented by saturation function $sat({u}_{\text{i}} )$, ${\text{i}} = 1, \ldots , {\mathrm{n}}$:

5 $$sat({u}_{\text{i}} ) = \left\{ {\begin{array}{*{20}l} {{u}_{\max }^{\text{i}} } & {{if}} & {{u}_{\text{i}} > {u}_{\max }^{\text{i}} } \\ {{u}_{\text{i}} } & {{if}} & {{u}_{\min }^{\text{i}} \le {u}_{\text{i}} \le {u}_{\max }^{\text{i}} } \\ {{u}_{\min }^{\text{i}} } & {{if}} & {{u}_{\text{i}} < {u}_{\min }^{\text{i}} } \\ \end{array} } \right.$$

where ${u}_{\max }^{\text{i}}$ (${u}_{\min }^{\text{i}}$) is the maximum (minimum) value of control input.

**Figure 1:**
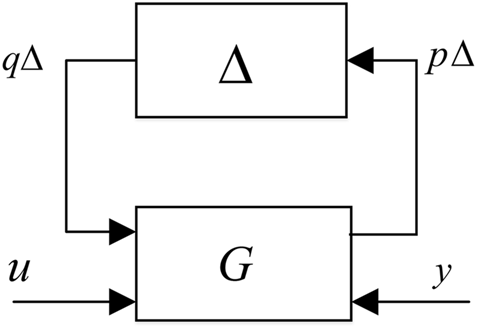
Plant model with structured norm-bounded uncertainty.

Assuming the plant as represented in Fig. 1, the input–output map from $u$ to $y$ of the plant $G(s)$ to be controlled can be modelled as

6 $$\left[ {\begin{array}{*{20}l} {p_{\Delta } } \\ y \\ \end{array} } \right] = \left[ {\begin{array}{*{20}l} {G_{11} (s)} & {G_{12} (s)} \\ {G_{21} (s)} & {G_{22} (s)} \\ \end{array} } \right]\left[ {\begin{array}{*{20}l} {q_{\Delta } } \\ u \\ \end{array} } \right]$$

7 $$q_{\Delta } = \Delta (s)p_{\Delta }$$

where $G_{22} (s)$ is the nominal plant, $G_{12} (s)$, $G_{21} (s)$ and $G_{11} (s)$ are known transfer function matrices that are used to describe the plant uncertainty in the frequency domain and the uncertainty is described by the element $\Delta (s)$, which satisfies the following norm inequality

8 $$\left\| {\Delta (s)p_{\Delta } } \right\|_{\Gamma }^{2} \le {{\left\| {p_{\Delta } } \right\|_{\Gamma }^{2} } \mathord{\left/ {\vphantom {{\left\| {p_{\Delta } } \right\|_{\Gamma }^{2} } {\gamma_{\Delta }^{2} }}} \right. \kern-\nulldelimiterspace} {\gamma_{\Delta }^{2} }},{\text{for}}\;{\text{all}}\quad p_{\Delta } \in L_{2}^{n} [0,\infty )$$

Here, $\Gamma$ belongs to some specified class of positive definite symmetric matrix and $\gamma_{\Delta }$ is some positive scalar^27^.

### Integral quadratic constraint notation and results

Under the IQC framework, the bounded operator $\Phi ( \cdot ):L_{2}^{{\text{n}}} \to L_{2}^{{\text{n}}}$ is said to satisfy the IQC defined by a bounded and self-adjoint operator $\Pi (s)$ or simply $\Phi ( \cdot ) \in IQC(\Pi )$ if the following inequality holds

9 $$\left\langle {\left[ {\begin{array}{*{20}l} p \\ q \\ \end{array} } \right],\Pi \left[ {\begin{array}{*{20}l} p \\ q \\ \end{array} } \right]} \right\rangle \ge 0,\quad \forall q = \Phi (p),\quad p \in L_{2}^{{\text{n}}}$$

where $\Pi (s):L_{2}^{{2{\text{n}}}} \to L_{2}^{{2{\text{n}}}}$ is an operator satisfying

10 $$\Pi (j\omega ) = \Pi^{*} (j\omega ),\quad \forall \omega$$

and $\left\langle {\cdot,\cdot } \right\rangle$ denotes the $L_{2}$ inner product^15^, *i.e.*

11 $$\left\langle {{f},{g}} \right\rangle = \frac{1}{2\pi }\int_{{{ - }\infty }}^{\infty } {{f}^{*} ({j}\omega ) \cdot {g}({j}\omega )} \cdot {\text{d}} \omega = \int_{0}^{\infty } {{\overset{\lower0.5em\hbox{$\smash{\scriptscriptstyle\frown}$}}{f} }^{*} ({t}){\overset{\lower0.5em\hbox{$\smash{\scriptscriptstyle\frown}$}}{g} }({t}){\text{d}} {t}}$$

here $${f}({s})$$ and $${g}({s})$$ denote the Fourier transforms of $${\overset{\lower0.5em\hbox{$\smash{\scriptscriptstyle\frown}$}}{f} }({t})$$ and $${\overset{\lower0.5em\hbox{$\smash{\scriptscriptstyle\frown}$}}{g} }({t})$$^26^.

**Figure 2:**
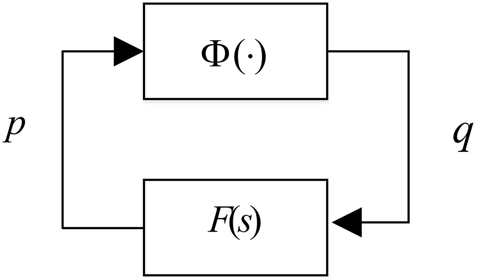
IQC set-up.

Consider the interconnection in Fig. 2,

12 $$q = \Phi (p),\quad p = F \cdot q,$$

where $\Phi (\cdot)$ is norm-bounded operator that encapsulates any nonlinearity or uncertainty in the loop satisfying (9). On the basis of IQC framework, reference^15,28^ presents the sufficient condition of stability for the interconnection in Fig. 2, namely.

#### **Lemma**

(IQC Sufficient Stability Condition)

*Assume that the upper left and lower right corner of *$\Pi (j\omega )$* are positive and negative semi-definite, respectively. In addition, assume the loop in question is well-posed, then stability of the interconnection in* Fig. 2*is guaranteed in the input–output sense provided that*^26^

13 $$\left[ {\begin{array}{*{20}l} {F(j\omega )} \\ {I_{{{\mathrm{n}} \times {\mathrm{n}}}} } \\ \end{array} } \right]^{ * } \Pi (j\omega )\left[ {\begin{array}{*{20}l} {F(j\omega )} \\ {I_{{{\mathrm{n}} \times {\mathrm{n}}}} } \\ \end{array} } \right] < 0\quad {\text{for all}}\,\omega .$$

### Structure of model following control

**Figure 3:**
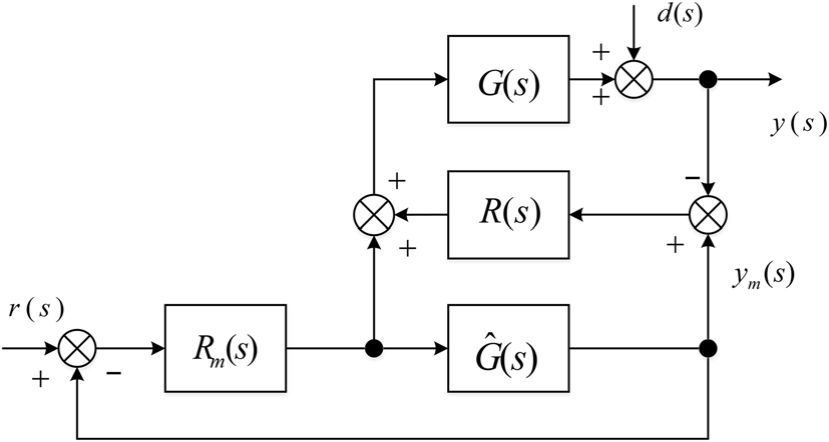
MFC structure.

As for SISO system, MFC structure is depicted in Fig. 3^23,24^, where symbols $G(s)$, $\hat{G}(s)$, $r(s)$ and $d(s)$ denote the plant, the reference model, the reference signal and the disturbance respectively. $R(s)$ and $R_{{\text{m}}} (s)$ are the correcting controller and the model controller.

Output $y(s)$ can be written as

14 $$y(s) = \frac{{G(s)[1 + R(s)\hat{G}(s)]}}{{\hat{G}(s)[1 + R(s)G(s)]}} \cdot y_{{\text{m}}} (s) + \frac{1}{1 + R(s)G(s)} \cdot d(s)$$

where $$y_{{\text{m}}} (s) = \frac{{R_{{\text{m}}} \hat{G}(s)}}{{1 + R_{{\text{m}}} (s)\hat{G}(s)}}r(s)$$.

When there is some perturbations $\Delta (s)$ in the plant, viz. $G(s) = \hat{G}(s)[1 + \Delta (s)]$, (10) can be rewritten as

15 $$y(s) = \left\{ {1 + \frac{\Delta (s)}{{1 + R(s)\hat{G}(s)[1 + \Delta (s)]}}} \right\} \cdot y_{{\text{m}}} (s) + \frac{1}{{1 + R(s)\hat{G}(s)[1 + \Delta (s)]}} \cdot d(s)$$

From (15), it is easy to get the relationship that is denoted by

16 $$y(s) \approx y_{{\text{m}}} (s) + \frac{1}{{1 + R(s)\hat{G}(s)[1 + \Delta (s)]}} \cdot d(s)$$

It is observed that the follow-up error $y(s) - y_{{\text{m}}} (s)$ does not depend on perturbations $\Delta (s)$ and the effect of disturbance can be eliminated by an appropriate adjusting of the correcting controller $R(s)$ ($R(s)$ is tuned to make $\left| {R(s)\hat{G}(s)} \right|$ large enough). And it shows that the MFC structure is very robust against both parameter variations and load disturbance of the actual plant^23,24^.

## Proposed anti-windup scheme based on MFC

**Figure 4:**
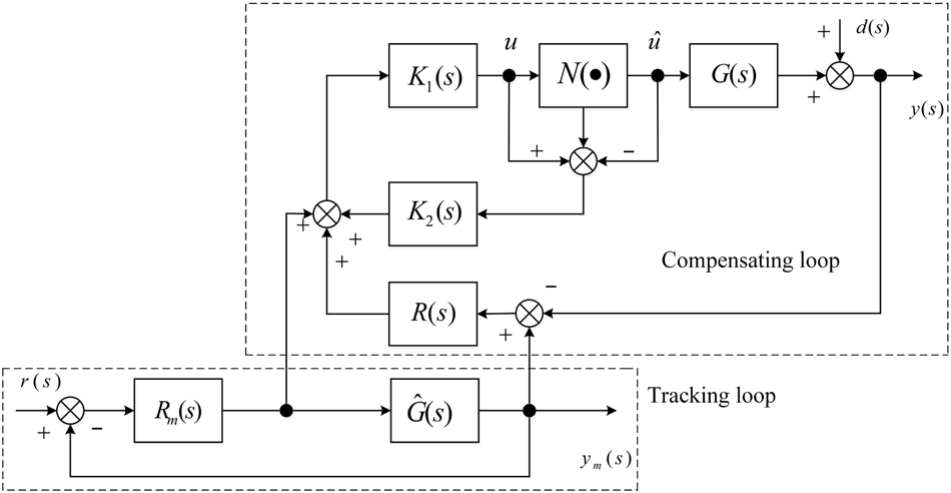
The proposed anti-windup scheme.

The structure diagram of the proposed MFC-based anti-windup scheme is shown in Fig. 4, where $K_{1} (s)$ and $K_{2} (s)$ are static or dynamic compensators; ${\mathcal{N}}(\cdot )$ denotes saturation nonlinearity, namely

17 $${\mathcal{N}}(\cdot ) = {diag}\{ sat({u}_{{i}} )\} ,\quad {\text{i}} = 1, \ldots , \mathrm{n}.$$

The proposed structure consists of two closed-loops, one is reference tracking loop and the other is feedback compensating loop. It should be noted that $R(s)$, $R_{{\text{m}}} (s)$, $K_{1} (s)$ and $K_{2} (s)$ can all be viewed as DoF’s (degree-of-freedom) for compensator synthesis. Thus, there are four DoF’s available for anti-windup compensation of the saturated system and hence noticeable performance improvement is expected.

In the real physical systems, the control input signal $u$ is always limited, thus it is reasonable to reformulate the saturation ${\mathcal{N}}(\cdot )$ for ${x}_{\mathrm{i}} \in {\mathbf{\mathbb{R}}}$ as:

18 $$sat({x}_{\mathrm{i}} ) = {N}_{\mathrm{i}} ({x}_{\mathrm{i}} ) \cdot {x}_{\mathrm{i}} ,\quad {\text{i}} = 1, \ldots , {\mathrm{n}}$$

or

$${\mathcal{N}}(\cdot ) = {N}({x}) \cdot {x,}\quad {N}({x}) = {diag}\{ {N}_{\mathrm{i}} ({x}_{\mathrm{i}} )\} ;$$

where ${N}_{\mathrm{i}} ({x}_{\mathrm{i}} )$ is some nonlinear function of variable ${x}_{\mathrm{i}}$. For some constant $\delta$ and every i (${\text{i}} = 1, \ldots , {\mathrm{n}}$), it is verified that

19 $$0 < \delta \le {N}_{\mathrm{i}} ({x}_{\mathrm{i}} ) \le 1,\quad \forall \left| {{x}_{\mathrm{i}} } \right| < \infty$$

20 $${N}_{\mathrm{i}}({x}_{\mathrm{i}} ) = 1\quad {\text{if}}\quad u_{\min }^{\mathrm{i}} \le x_{{\text{i}}} (t) \le u_{\max }^{\mathrm{i}} .$$

Hence, for some constant $\overline{\delta }$ and every ${\text{i}} = 1, \ldots , \mathrm{n}$, the inverse of diagonal matrix ${N}({x}) = {diag}\{ {N}_{\mathrm{i}} ({x}_{\mathrm{i}} )\}$ always exists:

21 $$1 \le {N}_{\mathrm{i}}^{ - 1} ({x}_{\mathrm{i}} ) = \frac{1}{{{N}_{\mathrm{i}}^{{}} ({x}_{\mathrm{i}} )}} \le \overline{\delta } = \frac{1}{\delta } < \infty ,\quad {\text{for\,any}}\quad \left| {{x}_{\mathrm{i}} } \right| < \infty$$

22 $${N}_{\mathrm{i}}^{ - 1} ({x}_{\mathrm{i}} ) = 1,\quad {u}_{\min }^{\mathrm{i}} \le {x}_{\mathrm{i}} ({t}) \le {u}_{\max }^{\mathrm{i}}$$

The system’s output $y(s)$ in Fig. 4 can be rewritten as

23 $$y(s) = H_{\mathrm{r}} (s) \cdot r(s) + H_{\mathrm{d}} (s) \cdot d(s)$$

where set-point transfer function $H_{\mathrm{r}} (s)$ is

24 $$H_{\mathrm{r}} (s) = G \cdot [(I_{{{\mathrm{n}} \times {\mathrm{n}}}} - K_{1} K_{2} )N^{ - 1} + K_{1} K_{2} + K_{1} RG]^{ - 1} \cdot K_{1} [I_{{{\mathrm{n}} \times {\mathrm{n}}}} + R\hat{G}][I_{{{\mathrm{n}} \times {\mathrm{n}}}} + \hat{G}R_{\text{m}} ]^{ - 1} R_{\mathrm{m}}$$

or

25 $$H_{\mathrm{r}} (s) = \frac{{K_{1} (s)R_{\text{m}} (s)G(s) \cdot [1 + R(s)\hat{G}(s)]}}{{[1 + R_{\text{m}} (s)\hat{G}(s)] \cdot [\frac{{{I}_{{{\mathrm{n}} \times {\mathrm{n}}}} - K_{1} (s)K_{2} (s)}}{N} + K_{1} (s)K_{2} (s) + K_{1} (s)R(s)G(s)]}}$$

for SISO systems, and load disturbance transfer function $H_{\text{d}} (s)$ is

26 $$H_{\mathrm{d}} (s) = \{ I_{{{\mathrm{n}} \times {\mathrm{n}}}} + G \cdot [(I_{{{\mathrm{n}} \times {\mathrm{n}}}} - K_{1} K_{2} )N^{ - 1} + K_{1} K_{2} ]^{ - 1} \cdot K_{1} R\}^{ - 1} .$$

or

27 $$H_{{\text{d}}} (s) = \frac{{K_{1} (s)K_{2} (s) + {{[1 - K_{1} (s)K_{2} (s)]} \mathord{\left/ {\vphantom {{[1 - K_{1} (s)K_{2} (s)]} N}} \right. \kern-\nulldelimiterspace} N}}}{{K_{1} (s)K_{2} (s) + K_{1} (s)R(s)G(s) + {{[1 - K_{1} (s)K_{2} (s)]} \mathord{\left/ {\vphantom {{[1 - K_{1} (s)K_{2} (s)]} N}} \right. \kern-\nulldelimiterspace} N}}}$$

for SISO systems.

When $K_{1} (s)K_{2} (s)$ is chosen to be approached to ${I}_{{{\mathrm{n}} \times {\mathrm{n}}}}$ (or 1 for SISO systems), namely

28 $$K_{1} (s)K_{2} (s) \to I_{{{\mathrm{n}} \times {\mathrm{n}}}} ,{\text{or}}\,K_{1} (s)K_{2} (s) \to 1\,({\text{SISO}}),$$

it follows (21), (22) that

29 $$[I_{{{\mathrm{n}} \times {\mathrm{n}}}} - K_{1} (s)K_{2} (s)] \cdot N^{ - 1} \to 0_{{{\mathrm{n}} \times {\mathrm{n}}}} \,{\text{or}}\,\frac{{1 - K_{1} (s)K_{2} (s)}}{N} \to 0\,({\text{SISO}}),$$

This means that effect of saturation nonlinearity ${\mathcal{N}}(\cdot )$ is almost eliminated; and hence $H_{{\text{r}}} (s)$ and $H_{{\text{d}}} (s)$ are approximately reformulated as

30 $$H_{\mathrm{r}} (s) = G \cdot [K_{2} + RG]^{ - 1} \cdot [I_{{{\mathrm{n}} \times {\mathrm{n}}}} + R\hat{G}][I_{{{\mathrm{n}} \times {\mathrm{n}}}} + \hat{G}R_{\text{m}} ]^{ - 1} R_{\text{m}}$$

31 $$H_{\mathrm{d}} (s) = [I_{{{\mathrm{n}} \times {\mathrm{n}}}} + G \cdot K_{2}^{ - 1} \cdot R]^{ - 1}$$

or

32 $$H_{\mathrm{r}} (s) = \frac{{R_{\text{m}} (s)G(s) \cdot [1 + R(s)\hat{G}(s)]}}{{[1 + R_{\mathrm{m}} (s)\hat{G}(s)] \cdot [K_{2} (s) + R(s)G(s)]}}$$

33 $$H_{\text{d}} (s) = \frac{1}{{1 + {{R(s)G(s)} \mathord{\left/ {\vphantom {{R(s)G(s)} {K_{2} (s)}}} \right. \kern-\nulldelimiterspace} {K_{2} (s)}}}}$$

for SISO systems. Therefore, according to (23) with (24), (25) or (26), (27), some attractive properties of the proposed anti-windup scheme are observed below:

### **Property-1**

$K_{1} (s)$ is almost decoupled from the behavior of the closed-loop system including set-point tracking and load-disturbance response when (28) is satisfied.

### **Property-2**

When (28) is valid, the set-point response and the disturbance response of the resulting anti-windup system are decoupled from each other. And besides, the disturbance response $H_{\text{d}} (s)$ is independent of $K_{1} (s)$, $R_{{\text{m}}} (s)$ and $\hat{G}(s)$.

### *Remark-1*

On the basis of *Property-1* and *Property-2*, there are some rules followed by compensator design:

(1) $K_{1} (s)$ is used to tune to satisfy (28);

(2) The correcting controller $R(s)$ is used to tune the performance of disturbance rejection while the model controller $R_{{\text{m}}} (s)$ is used to design the set-point response of the closed-loop system. ◊

### *Remark-2*

In the proposed anti-windup scheme, the nominal model $\hat{G}(s)$ can be regarded as a reference model, which means that $\hat{G}(s)$ is not necessarily identical to the real process $G(s)$. Therefore, $\hat{G}(s)$ can be chosen to exclude the unexpected characteristics of $G(s)$. ◊

Supposed that controller $R(s)$ and $R_{\text{m}} (s)$ are chosen as forms of PID controller:

34 $$R(s) = K + \frac{{T_{\text{i} } }}{s} + T_{\text{d} } s,K = diag\{ K^{\mathrm{j}} \} ,T_{\text{i} } = diag\{ T_{\mathrm{i}}^{\mathrm{j}} \} ,T_{\text{d} } = diag\{ T_{\text{d} }^{\mathrm{j}} \} ,{\mathrm{j}} = 1, \ldots , \mathrm{n}$$

35 $$R(s) = K + \frac{{T_{\text{i}} }}{s} + T_{\text{d}} s,K_{\text{m}} = diag\{ K_{\text{m}}^{{\text{j}}} \} ,T_{{{\text{i}} ,{\text{m}} }} = diag\{ T_{{i,{\text{m}} }}^{\mathrm{j}} \} ,T_{{{\text{d}} ,{\text{m}} }} = diag\{ T_{{{\text{d}} ,{\text{m}} }}^{\mathrm{j}} \} ,{\mathrm{j}} = 1, \ldots ,n$$

where $(K^{\mathrm{j}} ,\;T_{\mathrm{i}}^{\mathrm{j}} ,\;T_{d}^{\mathrm{j}} )$ and $(K_{{\mathrm{m}}}^{\mathrm{j}} ,\;T_{{\mathrm{i,m}}}^{\mathrm{j}} ,\;T_{{\mathrm{d,m}}}^{\mathrm{j}} )$ are parameters of *j*th PID controller. When reference signal $r(s)$ and load disturbance $d(s)$ are considered as step signal:

36 $$r(s) = \frac{1}{s}{\varvec{\alpha}} ,\quad {{\varvec{\upalpha}}} = [\alpha_{1} \;\;\alpha_{2} \;\; \ldots \;\;\alpha_{n} ]^{\mathrm{T}}$$

37 $$d(s) = \frac{1}{s}{{\varvec{\upbeta}}},\quad {{\varvec{\upbeta}}} = [\beta_{1} \;\;\beta_{2} \;\; \ldots \;\;\beta_{n} ]^{\mathrm{T}}$$

where ${{\varvec{\upalpha}}}$ and ${{\varvec{\upbeta}}}$ are constant vectors. Then, in a steady state, we have the following static relationship of the resulting closed-loop system

38 $$\overline{u} = - (I_{{{\mathrm{n}} \times {\mathrm{n}}}} + \overline{K}_{1} K\overline{G})^{ - 1} \overline{K}_{1} K \cdot {{\varvec{\upbeta}}}\quad {\text{for}}\quad r(s) = 0$$

39 $$\overline{u} = (I_{{{\mathrm{n}} \times {\mathrm{n}}}} + \overline{K}_{1} K\overline{G})^{ - 1} \overline{K}_{1} (I + K\overline{\hat{G}}) \cdot [I_{{{\mathrm{n}} \times {\mathrm{n}}}} + \overline{\hat{G}}K_{\text{m}} ]^{ - 1} K_{\text{m}} \cdot {{\varvec{\upalpha}}}\quad {\text{for}}\quad d(s) = 0$$

if the following condition is satisfied:

40 $$u_{\min }^{\mathrm{i}} \le \overline{u}_{\text{i}} \le u_{\max }^{\mathrm{i}} ,\quad {\text{i}} = 1, \ldots , \mathrm{n};$$

where $\overline{u}_{\mathrm{i}}$ is steady value of $u_{\mathrm{i}} (t)$; matrices $\overline{G}$, $\overline{K}_{1}$ and $\overline{\hat{G}}$ are the static gain matrix of transfer function $G(s)$, $K_{1} (s)$ and $\hat{G}(s)$ respectively. Furthermore, according to (24), (25) or (26), (27), we have

41 $$\mathop {\lim }\limits_{s \to 0} H_{\text{r}} (s) = I_{{{\mathrm{n}} \times {\mathrm{n}}}}$$

42 $$\mathop {\lim }\limits_{s \to 0} H_{\text{d}} (s) = 0_{{{\mathrm{n}} \times {\mathrm{n}}}}$$

### **Property-3**

When $R(s)$ and $R_{{\text{m}}} (s)$ are taken as PID controller like (34), (35), then the resulting anti-windup system can track the step reference signal without offset and reject the step load disturbance completely, namely

43 $$\mathop {\lim }\limits_{s \to 0} s \cdot H_{{{r}}} (s)\frac{1}{s}{{\varvec{\upalpha}}} = {{\varvec{\upalpha}}}$$

44 $$\mathop {\lim }\limits_{s \to 0} s \cdot H_{\mathrm{d}} (s)\frac{1}{s}{{\varvec{\upbeta}}} = 0$$

provide that

45 $${{\varvec{\upbeta}}} \in \Sigma_{\beta } \quad {\text{for}}\quad r(s) = 0$$

46 $${{\varvec{\upalpha}}} \in \Sigma_{\alpha } \quad {\text{for}}\quad d(s) = 0$$

where

47 $$\Sigma_{\beta } = \left\{ {\left. x \right|x \in - K\overline{K}_{1} (I_{{{\mathrm{n}} \times {\mathrm{n}}}} + \overline{K}_{1} K\overline{G}) \cdot z\,,\;z_{\mathrm{i}} \in [u_{\min }^{\mathrm{i}} ,\;u_{\max }^{\mathrm{i}} ]} \right\}$$

48 $$\Sigma_{\beta } = \left\{ {\left. x \right|x \in K_{\text{m}}^{ - 1} [I_{{{\mathrm{n}} \times {\mathrm{n}}}} + \overline{\hat{G}}K_{\text{m}} ] \cdot (I + K\overline{\hat{G}})^{ - 1} \cdot \overline{K}_{1}^{ - 1} (I_{{{\mathrm{n}} \times {\mathrm{n}}}} + \overline{K}_{1} K\overline{G}) \cdot z\,,\;z_{\mathrm{i}} \in [u_{\min }^{\mathrm{i}} ,\;u_{\max }^{\mathrm{i}} ]} \right\}$$

The above property can be easy to extend to the case of SISO systems.

When $\hat{G}(s)$ is viewed as reference model, output $y(s)$ in Fig. 4 can be reformulated by the reference output $y_{\text{m}} (s)$ and load disturbance *d*(*s*) as follows

49 $$y(s) = \tilde{H}_{\mathrm{r}} (s) \cdot y_{\text{m}} (s) + H_{\mathrm{d}} (s) \cdot d(s),$$

50 $$y_{\text{m}} (s) = [1 + \hat{G}(s)R_{\mathrm{m}} (s)]^{ - 1} \hat{G}(s)R_{\text{m}} \cdot r(s),$$

where $H_{\mathrm{d}} (s)$ is given in (26) (or SISO (27)), or (31) (or SISO (33)) approximately; the set-point transfer function $\tilde{H}_{\mathrm{r}} (s)$ is

51 $$\tilde{H}_{\mathrm{r}} (s) = \left\{ { R + [(K_{1}^{ - 1} - K_{2} )N^{ - 1} } \right. + K_{2} ] \cdot \left. {G^{ - 1} } \right\}^{ - 1} \cdot (\hat{G}^{ - 1} + R)$$

or, for SISO systems,

52 $$\tilde{H}_{\mathrm{r}} (s) = \frac{{RG + {G \mathord{\left/ {\vphantom {G {\hat{G}}}} \right. \kern-\nulldelimiterspace} {\hat{G}}}}}{{K_{2} + RG + {{(K_{1}^{ - 1} - K_{2} )} \mathord{\left/ {\vphantom {{(K_{1}^{ - 1} - K_{2} )} N}} \right. \kern-\nulldelimiterspace} N}}}$$

When (28) is valid, (51) or (52) can be rewritten as

53 $$\tilde{H}_{\mathrm{r}} (s) = [R + K_{1}^{ - 1} G^{ - 1} ]^{ - 1} \cdot (\hat{G}^{ - 1} + R)$$

54 $$\tilde{H}_{\mathrm{r}} (s) = \frac{{RG + {G \mathord{\left/ {\vphantom {G {\hat{G}}}} \right. \kern-\nulldelimiterspace} {\hat{G}}}}}{{K_{1}^{ - 1} + RG}} \left( {{\text{SISO}}} \right)$$

According to (53), (54) and (31), (33), it is verified that:

Therefore, the following property of the proposed anti-windup system is obtained,

### **Property-4**

In mode of reference control (49), (50), both ${K}_{1} ({s})$ are ${K}_{2} ({s})$ are almost decoupled from the reference output signal $y_{\text{m}} (s)$ in (50). When (28) is satisfied, the load-disturbance response is independent of ${K}_{1} ({s})$, $R_{{\text{m}}} (s)$ and $\hat{G}(s)$, and the tracking response is independent of ${K}_{2} ({s})$ and $R_{{\text{m}}} (s)$. Furthermore, *Property -3* is still valid when (45), (46) is satisfied.

## Robustness analysis

IQC-based (Integral Quadratic Constraints) approach receives many attentions in robust control, many classical robust control tools and concepts such as Small Gain, Circle Criterion, Popov Criterion and the Zames-Falb multiplier can be conveniently expressed by IQCs. The IQC theory also provides a framework (Fig. 2) for combining plant uncertainties and nonlinearities for both robust analysis and synthesis^1,15,28^. Based on the IQC sufficient stability condition, this section provides a robustness guarantee (Theorem) for the MFC-based anti-windup scheme against norm-bounded uncertainty expressed in the more general Linear Fractional Transformation form (Fig. 3).

Recalling the IQC notation and results, as pointed out by^1^, when the bounded operator $\Phi (\cdot)$ is considered as dead-zone nonlinearity, namely

55 $$\Phi (p_{{{{dz}}}} ) = {dz}(p_{{{{dz}}}} ),$$

56 $${dz}( \cdot ) = [{dz}_{1} ( \cdot )\;\,{dz}_{2} ( \cdot )\; \ldots \;{dz}_{\text{n}} ( \cdot )]^{\rm T} ,$$

then $\Phi ( \cdot ) = {dz}( \cdot ) \in IQC(\Pi_{1} )$ is valid , in which $\Pi_{1} (s)$ is formulated by a class of admissible function $W(s)$:

57 $$\Pi_{1} = \left[ {\begin{array}{*{20}l} 0 & {W^{*} (s)} \\ {W(s)} & { - W(s) - W^{*} (s)} \\ \end{array} } \right],$$

where ${dz}_{i} ( \cdot )$ represents the dead-zone function.

When the bounded operator $\Phi ( \cdot )$ is considered as norm-bounded uncertainty, namely

58 $$\Phi (p_{\Delta } ) = \Delta (p_{\Delta } ),$$

59 $$\Delta ( \cdot ) = [\Delta _{1} ( \cdot )\Delta _{2} ( \cdot ) \cdots \Delta _{{\text{n}}} ( \cdot )]^{{\text{T}}} ,$$

then $\Phi ( \cdot ) = \Delta ( \cdot ) \in IQC(\Pi _{2} )$ is valid, in which $\Pi _{2} (s)$ is formulated by some specified class of positive definite symmetric matrix $\Gamma (s)$ and some positive scalar $\gamma _{\Delta }$:

60 $$\Pi _{2} = \left[ {\begin{array}{*{20}c} {\Gamma (s)} & 0 \\ 0 & { - \gamma _{\Delta }^{2} \Gamma (s)} \\ \end{array} } \right],$$

According to (57), (60), we have $$\Phi ( \cdot ) = \left[ {\begin{array}{*{20}l} {dz( \cdot )} & \\ & {\Delta ( \cdot )} \\ \end{array} } \right] \in IQC(\Pi )$$ with

61 $$\Pi = \left[ {\begin{array}{*{20}c} 0 & 0 & {W^{*} (s)} & 0 \\ 0 & {\Gamma (s)} & 0 & 0 \\ {W(s)} & 0 & { - W(s) - W^{*} (s)} & 0 \\ 0 & 0 & 0 & { - \gamma _{\Delta }^{2} \Gamma (s)} \\ \end{array} } \right]$$

It is easy to verify that

62 $${dz}(x) = x - {sat}(x) .$$

**Figure 5:**
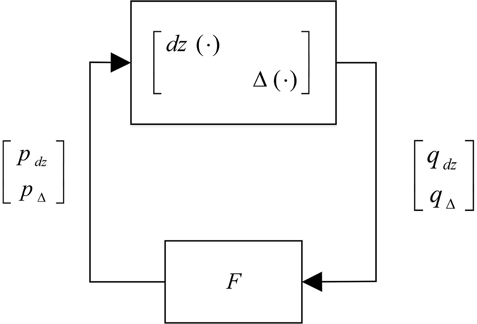
Equivalent interconnection of the compensating loop in the proposed scheme.

Therefore, ignoring the reference signal $r(s)$ and the exogenous disturbance $d(s)$, the compensating loop in Fig. 4 can be transformed into the feedback interconnection in Fig. 5. It can be taken as special case of (9) in Fig. 2, where

63 $$p = \left[ {\begin{array}{*{20}c} {p_{{{\mathrm{dz}}}} } \\ {p_{\Delta } } \\ \end{array} } \right],q = \left[ {\begin{array}{*{20}c} {q_{{{\mathrm{dz}}}} } \\ {q_{\Delta } } \\ \end{array} } \right],\Phi ( \cdot ) = \left[ {\begin{array}{*{20}c} {{dz}( \cdot )} & {} \\ {} & {\Delta ( \cdot )} \\ \end{array} } \right],$$

64 $$F = \left[ {\begin{array}{*{20}c} {K_{1} (s)K_{2} (s) - H(s)G_{{22}} (s) \cdot [I_{{{\mathrm{n}} \times {\mathrm{n}}}} - K_{1} (s)K_{2} (s)]} & { - H(s)G_{{21}} (s)} \\ {[G_{{12}} (s)H(s)G_{{22}} (s) + G_{{12}} (s)] \cdot [I_{{{\mathrm{n}} \times {\mathrm{n}}}} - K_{1} (s)K_{2} (s)]} & {G_{{11}} (s) + G_{{12}} (s)H(s)G_{{21}} (s)} \\ \end{array} } \right],$$

65 $$H(s) = - [I_{{{\mathrm{n}} \times {\mathrm{n}}}} + K_{1} (s)R(s)G_{{22}} (s)]^{{ - 1}} \cdot K_{1} (s)R(s),$$

By straight application of *Lemma*, we have the following robustness criterion for the resulting system of the proposed MFC/PID-based anti-windup scheme.

### **Theorem**

The closed-loop system of the proposed MFC/PID-based anti-windup scheme in Fig. 4 is stable provided that.

(1) Model controller $R_{{\text{m}}} (s)$ makes the reference tracking loop stable;

(2) $K_{{{1}}} (s)$, $K_{{{2}}} (s)$, $R(s)$ and $W(s)$ are chosen such that $L_{S} (j\omega ) < 0$ for all $\omega$ where

66 $$L_{S} = \left[ {\begin{array}{*{20}c} {L_{{11}} } & {L_{{21}}^{*} } \\ {L_{{21}} } & {L_{{22}} } \\ \end{array} } \right]$$

and

67 $$\begin{aligned} L_{{11}} & = \Theta _{1}^{*} (s)G_{{12}}^{*} (s)\Gamma (s)G_{{12}} (s)\Theta _{1} (s) - \Theta _{1}^{*} (s)W^{*} (s) - W(s)\Theta _{1} (s) \\ L_{{21}} & = - G_{{21}}^{*} (s)H^{*} (s)W^{*} (s) + \Theta _{2}^{*} (s)\Gamma (s)G_{{12}} (s)\Theta _{1} (s) \\ L_{{22}} & = \Theta _{2}^{*} (s)\Gamma (s)\Theta _{2} (s) - \gamma _{\Delta }^{2} \Gamma (s) \\ \end{aligned}$$

and

68 $$\begin{aligned} \Theta _{1} (s) & = [I_{{{\mathrm{n}} \times {\mathrm{n}}}} + H(s)G_{{22}} (s)] \cdot [I_{{{\mathrm{n}} \times {\mathrm{n}}}} - K_{1} (s)K_{2} (s)] \\ \Theta _{2} (s) & = G_{{11}} (s) + G_{{12}} (s)H(s)G_{{21}} (s) \\ \end{aligned}$$

## Numerical example

To demonstrate the implications of our results we use the academic example introduced in^29^. Numerical simulations for tracking (amplitude of the pulse reference input signal is 1.2) are conducted by robustness comparison among four different anti-windup schemes, viz. IMC-based scheme^8^, static anti-windup compensation^8^ high-gain anti-windup compensation^10^ and dynamic compensation by Weston & Postlethwaite^13^. It shows that IMC-based scheme can achieve better robustness than the three ones in the case that the following plant $G(s)$ (which has a large resonant peak) with controller $C(s)$ is considered as follows

69 $$G(s) = \frac{{10}}{{s^{2} + 0.01s + 10}},u_{{\max }} = 1,u_{{\min }} = - 1$$

and its state-space realization70

Therefore, simulation is to take a comparison of robustness between the proposed scheme and four other schemes viz. IMC-based, static, high-gain and Weston & Postlethwaite’s compensation scheme. It should be emphasized that controller $C(s)$ of the later four schemes is identical and given as follows71
viz.

72 $$C_{1} (s) = \frac{{337.5s^{2} + 3375s + 8437.5}}{{s^{3} + 82.5s^{2} + 200s}},C_{2} (s) = - \frac{{135s^{3} + 1687.5s^{2} + 6750s + 8437.5}}{{s^{3} + 82.5s^{2} + 200s}}$$

Compensators designed by^8^ for static, high-gain, IMC-based and Weston & Postlethwaite’s compensation scheme are recalled below,

(I) *Static anti-windup compensation.*

**Figure 6:**
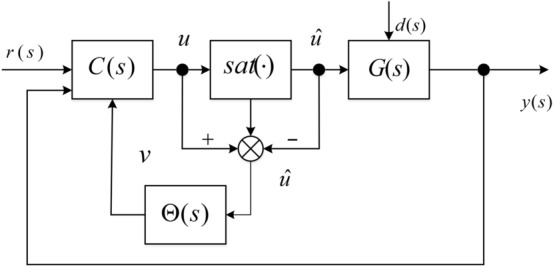
Structure of static antiwindup scheme.

The diagram is displayed in Fig. 6, where the static anti-windup compensator is computed as

73 $$\Theta = \left[ {\begin{array}{*{20}c} { - 0.1909} \\ {0.1402} \\ \end{array} } \right].$$

(II) *High-gain anti-windup compensation.*

In terms of Fig. 6, the high-gain anti-windup compensator is chosen as

74 $$\Theta = \left[ {\begin{array}{*{20}c} 0 \\ {14} \\ \end{array} } \right].$$

(III) *Weston and Postlethwaite’s scheme of anti-windup compensation.*

**Figure 7:**
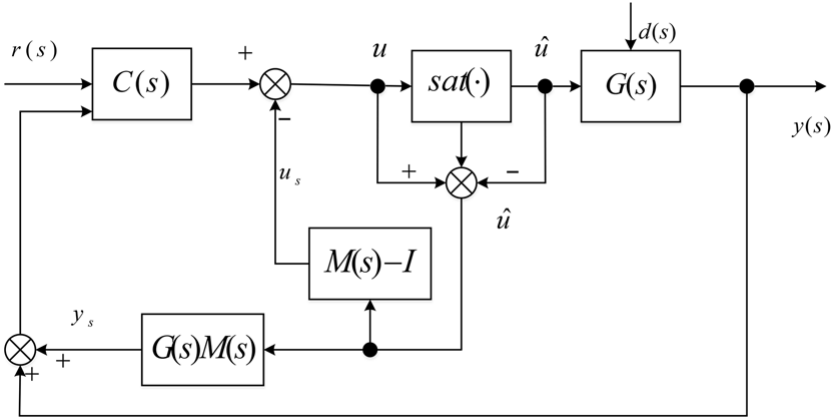
Anti-windup scheme proposed by Weston & Postlethwaite.

The diagram is displayed in Fig. 7, $M(s)$ is chosen as75

(IV) *IMC anti-windup scheme.*

IMC anti-windup scheme can be viewed as a special case of Weston & Postlethwaite’s compensation scheme, where $M(s) = I$.

(V) *The proposed anti-windup scheme.*

Therefore, according to the sufficient conditions in Theorem, ${{k}}_{1}$, $k_{2}$, $R(s)$ and $R_{{\text{m}}} (s)$ in the proposed scheme are chosen as follows

76 $$k_{1} = 0.21,k_{1} = 4.5,\hat{G}(s) = G(s);$$

77 $$R(s)\sim K = 18.1,T_{i} = 0.13,T_{d} = 4.06;$$

78 $$R_{\mathrm{m}} (s)\sim K_{m} = 1.8,T_{{i,m}} = 2.0,T_{{i,d}} = 0;$$

**Figure 8:**
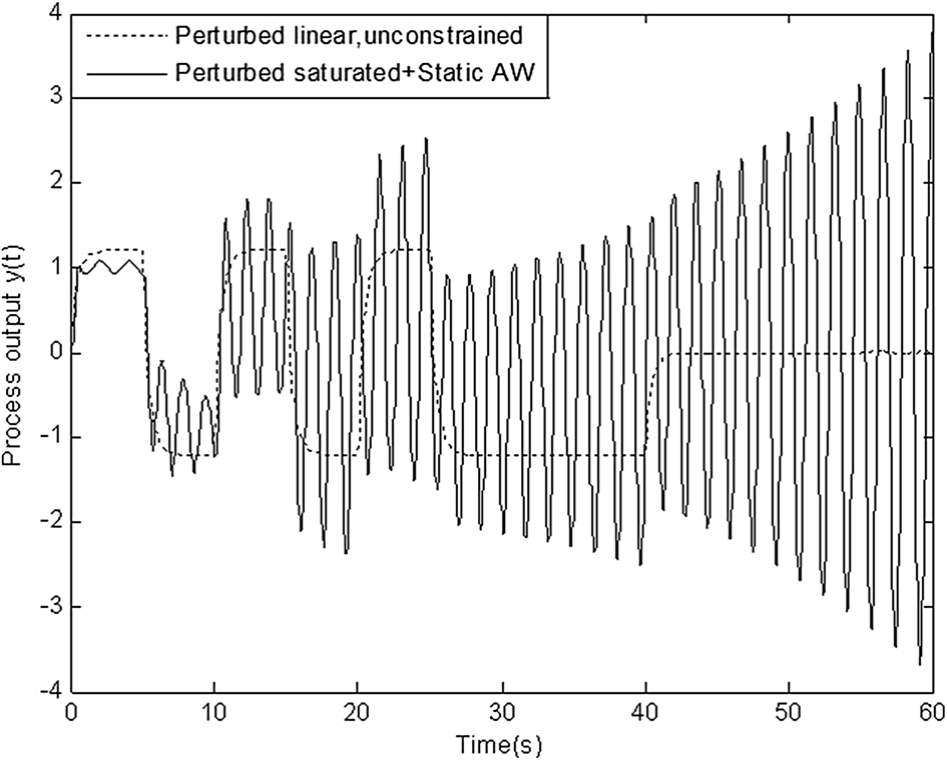
Response of perturbed system with static anti-windup.

**Figure 9:**
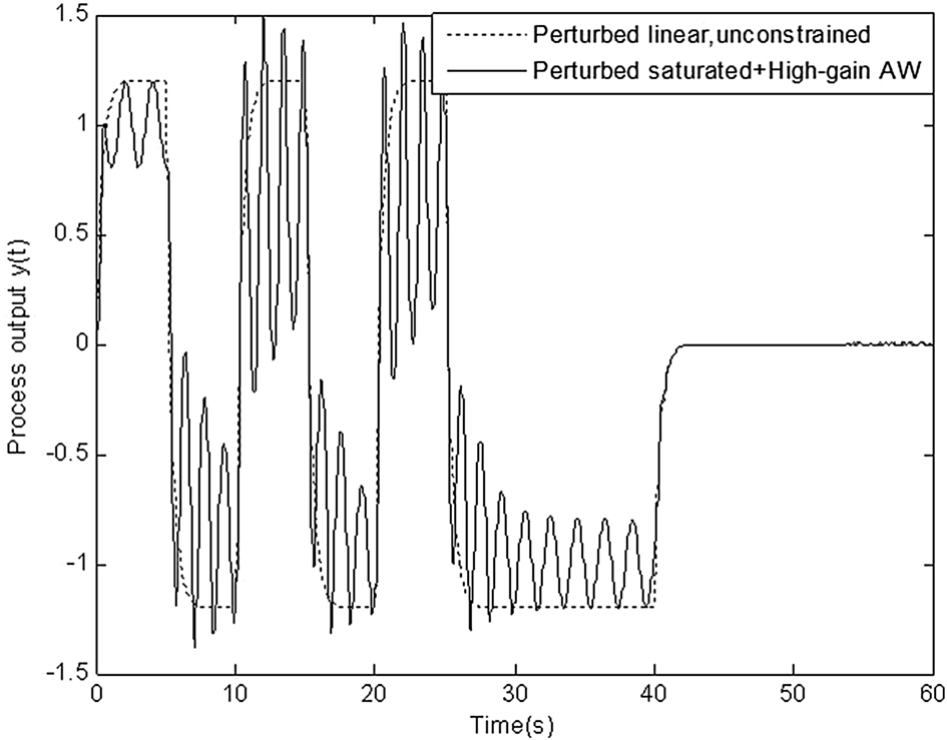
Response of perturbed system with high-gain anti-windup.

**Figure 10:**
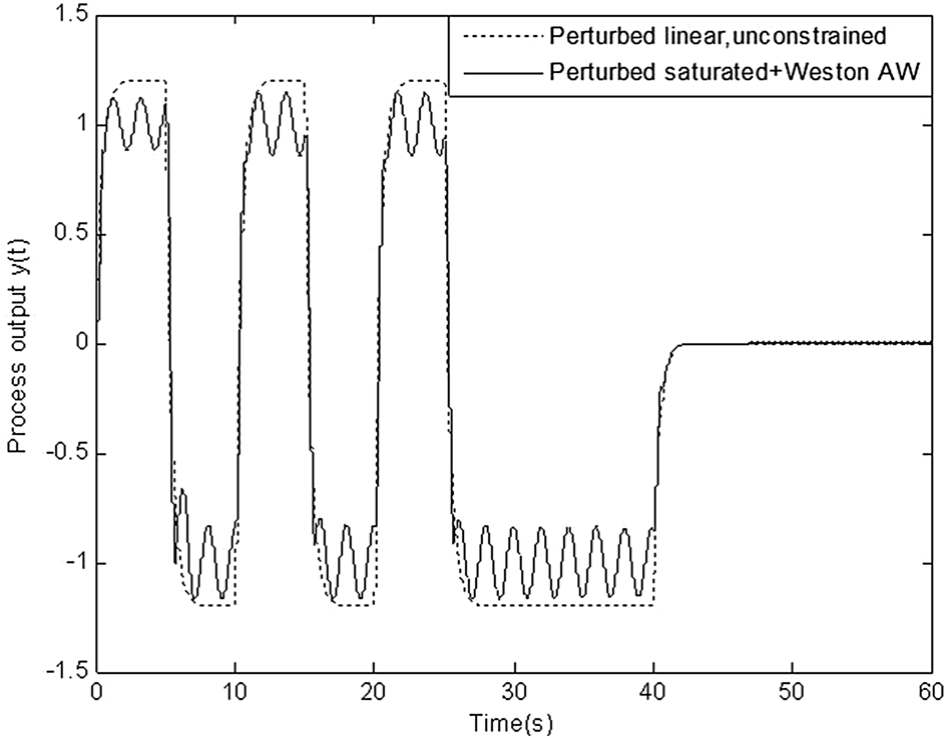
Response of perturbed system with Weston & Postlethwaite’s anti-windup.

**Figure 11:**
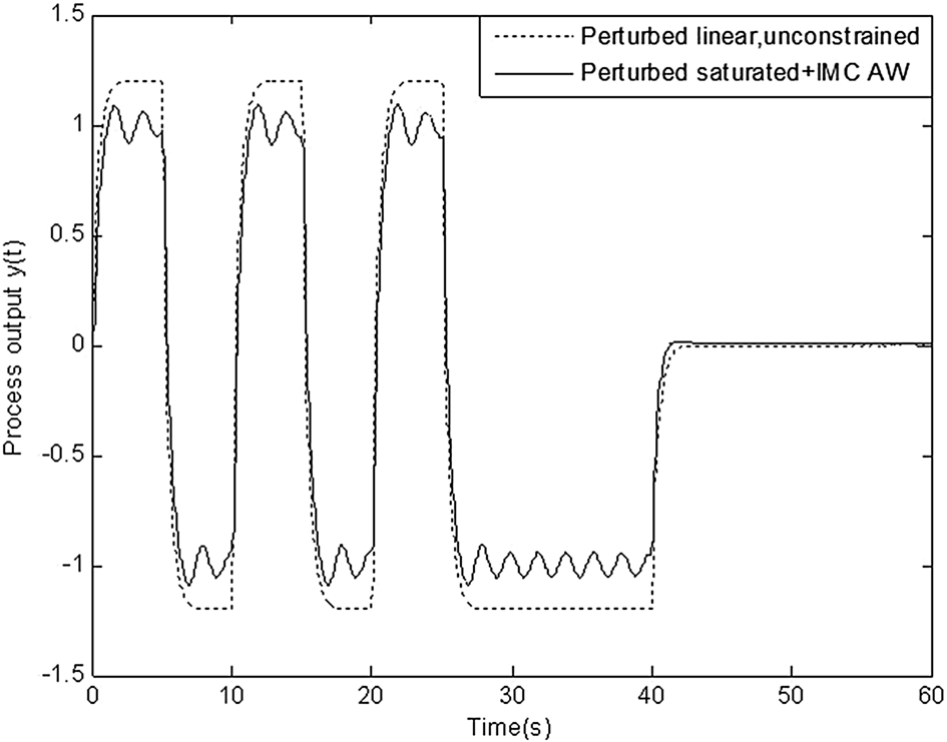
Figure 9 Response of perturbed system IMC anti-windup.

**Figure 12:**
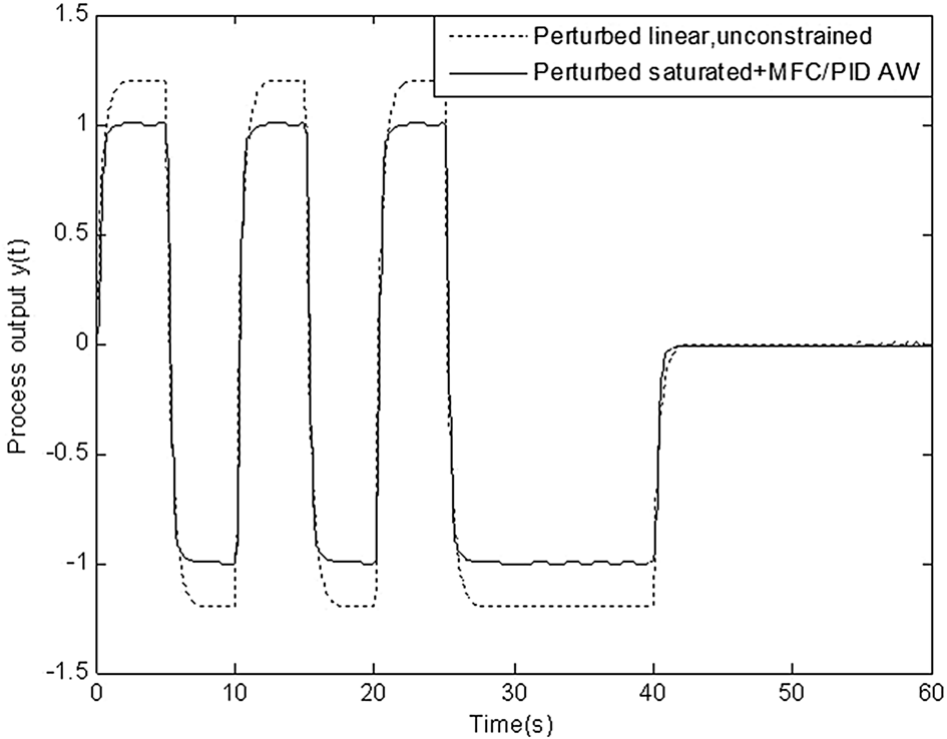
Figure 9 Response of perturbed system with MFC/PID robust anti-windup.

In this section, simulation is conducted to compare robustness of the proposed scheme with that of four schemes above. Figures 8, 9, 10, 11 and 12 show the response of perturbed system with static, high-gain, IMC, Weston & Postlethwaite’s anti-windup scheme and the proposed MFC/PID robust anti-windup scheme respectively. As we can see, robustness of the proposed scheme is obviously superior to that of others.

## Conclusions

This paper proposes a 4-degree-of-freedom (DoF) anti-windup scheme for the control system with actuator saturation and parametric uncertainty, which provides a more practical method by considering the plant with norm bounded uncertainty. Especially, due to four DoFs of the proposed scheme, set-point tracking response and load disturbance response can be designed separately in the resulting closed-loop system which can be specified arbitrarily in some sense. By using the IQC framework and its related lemma, a sufficient robust stability condition of the proposed anti-windup scheme is derived with considering the norm-bounded uncertainties of the plant. As a result, a fairly straightforward stability tuning rule to design the anti-windup compensators is obtained accordingly in the frequency domain. The effectiveness and the remarkable superior performance on set-point tracking and load-disturbance rejection of the proposed methods are demonstrated by carrying out the comparison of other anti-windup schemes like Static, High-Gain, IMC-based and Weston & Postlethwaite’s compensation scheme.
