# Author Correction: Robust MFC anti-windup scheme for LTI systems with norm-bounded uncertainty

**DOI:** 10.1038/s41598-021-89265-6

**Published:** 2021-04-29

**Authors:** Xiao-Qin Mo, Mi Zhou, Yuan Wang, Shang-Jia Guo

**Affiliations:** 1 Institute of Science and Technology, University of Sanya, Sanya City, 572000 Hainan Province China; 2 Haikou Power Supply Bureau, Hainan Power Grid Company, Haikou City, 570100 Hainan Province China

Correction to: *Scientific Reports* 10.1038/s41598-021-81036-7, published online 21 January 2021

This Article contains an error. Funding section was originally not included – it should appear as below:

